# Topical beta‐blockers in dermatologic therapy

**DOI:** 10.1111/dth.15016

**Published:** 2021-06-21

**Authors:** Angela Filoni, Francesca Ambrogio, Aurora De Marco, Alessia Pacifico, Domenico Bonamonte

**Affiliations:** ^1^ Section of Dermatology, Department of Biomedical Science and Human Oncology University of Bari Bari Italy; ^2^ Section of Dermatology Perrino Hospital Brindisi Italy; ^3^ Phototherapy Unit San Gallicano Dermatological Institute Rome Italy

**Keywords:** beta‐blockers, hemangiomas/vascular tumors, Kaposi, paronychia, therapy‐topical, wounds

## Abstract

An increasing use of beta‐blockers in dermatology has been described over the last 10 years, despite the fact that their use in diseases other than infantile hemangiomas is off‐label. This review discusses the emerging role of topical beta‐blockers in the treatment of infantile hemangioma, but also pyogenic granuloma, Kaposi sarcoma, wounds and nail paronychia. Data in literature demonstrate that topical beta‐blockers are a safe and valid therapeutic option in numerous cutaneous diseases. Side effects are mainly restricted to the application site. Further studies and randomized trials may contribute to reinforce the role of topical beta‐blockers in the dermatological armamentarium.

## 1 INTRODUCTION

An increasing use of beta‐blockers in dermatology has been described over the last 10 years, despite the fact that their use in diseases other than infantile hemangiomas is off‐label. Beta‐blockers antagonize the effects of circulating catecholamines on beta‐adrenoceptors; within the skin, these receptors are present on keratinocytes, fibroblasts, and melanocytes, so this wide distribution can justify their emerging role, for both topical and systemic use, in a multitude of dermatologic conditions.^1^, ^2^

The effects of beta‐blockers on infantile hemangiomas were discovered by chance; since then, many studies have been published demonstrating the efficacy and safety of propranolol in the treatment of infantile hemangiomas.^1^ The US Food and Drug Administration has recently approved oral propranolol for use in severe infantile hemangiomas.^1^

The present review discusses the emerging role of topical beta‐blockers in dermatology.

### 1.1 Literature search

All original articles and clinical trials present in PubMed database evaluating topical beta blockers in infantile hemangiomas treatment were included; instead case reports, and small studies (with less than five patients) on the treatment of infantile hemangiomas with topical beta blockers were not analyzed given the presence of numerous larger studies on this subject.

Concerning Kaposi sarcoma (KS), pyogenic granuloma (PG), nail paronychia, and wound management were analyzed not only original research articles, but also case studies and small studies (with fewer than five patients) given the few literature on these topics.

We have done also a literature review using the following search items “skin” or “skin disease” and “β‐blocker” in PubMed database to include other diseases than the ones analyzed above.

We have also discussed insights from review articles and meta‐analyses concerning topical beta blockers use in the overmentioned diseases to make a more comprehensive literature analysis.

Eligibility criteria for all the manuscripts analyzed were English language, availability of full text, and publications between January 2016 and July 2020. Articles not reporting a clinical outcome were excluded.

## 2 BETA‐BLOCKERS IN INFANTILE HEMANGIOMA TREATMENT

Infantile hemangiomas (IHs) are the most common pediatric vascular tumors.^1^ Despite their benign nature, IHs may have serious consequences, possibly affecting both the aesthetic and the functional outcomes, besides potentially being dramatic life‐threatening conditions.^1^

Oral propranolol (a non‐selective beta‐blocker) is currently considered as the first‐line agent for IHs requiring systemic treatment; this clinical use in IHs is based on randomized controlled trials showing a 60% successful treatment rate compared with 4% for patients receiving placebo.^1^

Many possible mechanisms of action have been postulated, mainly including vasoconstriction and decreased angiogenesis through the inhibition of both vascular endothelial growth factor (VEGF) and basic fibroblast growth factor. Additionally, propranolol stimulates apoptosis of endothelial cells, hemangioma‐derived stem cells, and pericytes.^1^, ^2^

Topical beta‐blockers are currently considered as an interesting alternative to oral administration for superficial thin hemangiomas. The most common topical beta‐blockers used in IHs are propranolol and timolol. Although topical timolol maleate appears to be more widely used compared to topical propranolol due to its already known use in ophthalmology, a meta‐analysis concluded that there was no significant difference between topical timolol and topical propranolol in treating IHs.^1^

Even if topical beta blockers may display an effective alternative to oral ones in treating superficial IHs, it is not possible to exclude that even topical beta blockers may be systemically absorbed, thus leading to the possible risk for side effects. Weibel et al. measured the systemic absorption throughout timolol detection both in urine and blood samples. Interestingly, although detectable levels of timolol were present in all samples, no systemic adverse effects were reported, thus suggesting that the systemic absorption of the drug does not reach potentially dangerous levels.^2^

Borok et al. in 2018 carried out a prospective study in order to explore possible risk factors for systemic absorption of 0.5% gel forming solution (GFS) topical timolol, as well as for eventual correlations with adverse effects. According to this study, patients treated with a higher daily dosage of topical timolol (two drops twice daily compared to 1 drop twice daily) display a higher risk for systemic absorption, especially when treated for scalp hemangiomas, although no correlations between timolol blood levels and adverse effects were found.^3^

However, although topical beta blockers have proved themselves effective and generally safe in otherwise‐healthy patients, their exact skin concentration cannot be precisely assessed. This has indeed generated many different speculations, as a non‐defined concentration in the affected site may lead to insufficient or else dangerously high drug levels, especially in potentially‐at‐risk patients. As a matter of fact, after analyzing 103 children treated with 0.5% topical timolol (both solution and GFS), Frommelt et al. advised against the use of topical timolol in premature patients, especially when administered at higher dosage than the recommended daily one of 0.25 mg/kg.^4^ Moreover, since timolol is metabolized by the cytochrome P450 2D6, it is possible that children with low cytochrome P450 2D6 activity levels may be at risk for overdosage.^5^

### 2.1 Topical beta blockers and topical propranolol formulations

As for timolol, the most common administered formulations are 0.5% eyedrops solution and 0.5% GFS, and even if they seem to produce similar clinical responses, eyedrops may be a more cost‐effective choice.^6^

However, since 0.5% topical timolol appears to be the most widely used formulation,^2^, ^7^, ^8^ a meta‐analysis conducted by Ng et al. in 2016, focused more extensively on the therapeutic protocols rather than on the concentrations, showing that encouraging results are indeed possible with different 0.5% topical timolol administration protocols (1 drop BID, 2 drops BID, 3 drops TID/QID, 3 drops TID), as Global Assessment Score greater than 3 (which indicates an acceptable clinical improvement) was registered in 47% up to 88% of cases.^9^

On the other hand, concerning topical propranolol, many different topical preparations have been used in the clinical practice, mainly including 1% propranolol gel, 2% propranolol cream,^9^ and 4% propranolol gel.^10^ The results, in all cases and for all the examined concentrations, are indeed encouraging as a relevant clinical improvement (from partial to excellent) was registered in 147 out of 148 patients treated with 1% propranolol gel twice daily for 12 weeks, as well as in 23 out of 40 patients treated with 2% propranolol cream three times per day,^11^ and even in 62 out of 75 patients treated with 4% propranolol gel twice daily.^10^

As a matter of fact, as reviewed by Price et al. in 2018 over a total of 632 hemangiomas, a significant clinical improvement with only mild local side effects was registered in 90% of cases, after topical propranolol was administered at very different concentrations, shifting from 0.5% to 5%.^12^

However, optimizing topical propranolol administration is always a challenging issue in the clinical practice, as not only the concentration but also the formulation may influence the drug permeation, absorption and even delivery to the affected site. In particular, Casiraghi et al., in 2016, focused upon four types of semi‐solid topical 1% propranolol preparations (a hydrophobic ointment, two lipophilic creams and a hydrophilic cream), thus showing that the highest levels of propranolol permeation are guaranteed by the hydrophilic cream while hydrophobic ointments may be not adequate for the purpose.^13^ However, a more recent work on topical propranolol, comparing ointment, creamy preparations and gel formulations, suggests that gel formulations may lead to better skin permeation profiles.^14^

### 2.2 Topical beta‐blockers versus placebo or versus oral beta‐blockers in hemangiomas

0.5% topical timolol demonstrated a 91% resolution rate in pooled meta‐analysis with a significantly greater percentage of improvement compared with the placebo group. Moreover, two recent meta‐analyses by Zheng and by Lin clearly assessed once again 0.5% topical timolol greater efficacy and safety compared to simple observation.^7^, ^8^

Topical beta blockers may also display an interesting role in the treatment of lesions in delicate sites, such as the head‐and‐neck area and the periocular one in particular, as demonstrated by many studies.^15^

In 2016 the Haemangioma Investigator Group reviewed over 700 cases of IHs treated with 0.5% GFS topical timolol, demonstrating both that after 9 months, topical timolol had induced a 30% improvement in the size of superficial, relatively thin IHs, and that topical timolol response is more gradual and modest compared to the one registered with oral beta‐blockers.^5^

On the other hand, in a recent meta‐analysis Lin et al. focusing upon the efficacy of both topical and oral beta blocker therapy in treating superficial IHs, assessed that no difference was found between topical propranolol or topical timolol and oral propranolol.^8^

According to a massive review of the literature published in 2018 by Novoa et al.^1^ there were no differences between 1 mg/kg/die oral propranolol and 0.5% topical timolol regarding their ability to induce a 50% or greater reduction in lesion size, although the quality of evidence was low. A systematic review of over 700 superficial IHs reported no significant differences in the clinical improvement between topical 0.5% timolol maleate hydrogel (three times a day) and oral propranolol (2 mg/kg/day), including cases characterized by the presence of IHs larger than 5 cm^2^. Interestingly, the incidence of systemic adverse events (AEs) between the two groups showed no significant differences, even if no systemic AEs were detected during topical timolol treatment, compared with 14 patients who experienced systemic AEs during oral propranolol treatment. However, mild local side effects were observed in 12 patients treated with timolol maleate 0.5% hydrogel, including local pruritus and skin blemishes.^16^

The risk for adverse effects; however, is still a quite debated issue; as a matter of fact, Lin et al. with an impotent meta‐analysis of the literature, assessed that there was no significant difference in the frequency of adverse effects caused by topical propranolol and oral one.^8^ On the other hand, it is possible to say that the severity of the adverse effects caused by oral beta blockers and topical ones is quite different. Topical timolol and propranolol are mainly known to cause local adverse effects such as eczema, ulcers, skin rashes, desquamation and erythema, while oral propranolol may induce gastrointestinal disorders, sleep disorders, bradycardia, hypotension, hypoglycemia, and even wheezing.^8^ Therefore, although topical beta blockers may induce adverse effects with the same frequency as for oral ones, it possible to assume that topical timolol and propranolol are generally safe, as they mainly cause manageable localized side effects.

However, even if no clear difference has been reported between oral and topical beta blockers in the treatment of infantile hemangiomas, a combination of both oral and topical formulations has been studied to treat compound hemangiomas, showing great results. Ge J. et al., in 2016, conducted indeed a retrospective study including 89 patients with compound hemangiomas treated both with oral propranolol (2 mg/kg/day, 1 mg twice daily) and topical timolol (0.5% timolol maleate gel three times per day) for at least 3 months. Therefore, according to this study, the combination of oral propranolol and topical timolol was proved to be effective, as it produced a clinical improvement in 89 out of 89 patients and a complete clinical resolution in 19 out of 89 patients, with only mild side effects (cold extremities, diarrhea, agitation during sleep) that did not require a discontinuation of the therapy.^17^

Moreover, another study compared a 14‐patient‐experimental group treated using oral propranolol (1 mg/kg/die) in combination with topical timolol maleate (0.5% eye drops, twice daily), whit a 17‐patient‐control group, treated using oral propranolol only (1.5 mg/kg/die). In this case, the experimental group showed a greater improvement in color compared to the control group, while no significant difference was noticed between the two groups in terms of both volume reduction and side effects (never severe, anyway). However, interestingly, the mean treatment duration in the experimental group resulted to be significantly shorter than the one reported in the control group, thus suggesting a possible role for topical beta blockers in shortening oral therapies.^18^

As a matter of fact, Mannschreck et al., in 2019, reported indeed a retrospective study analyzing five different groups of patients, depending on the therapeutic regimen they were on (propranolol only, timolol only, propranolol to timolol, timolol to propranolol to timolol, and timolol to propranolol). Interestingly, the patients treated with oral propranolol followed by topical timolol (0.5% GFS; one drop twice daily) underwent the shortest duration of oral propranolol therapy, thus reinforcing the idea of using topical beta blockers both in monotherapy to treat above all superficial IHs, and in combination with oral propranolol to reduce and improve the overall therapy for compound lesions.^19^

### 2.3 Topical beta blockers versus other therapeutic options in hemangiomas

Many studies compared topical beta blockers use with more some of the therapies previously used to treat IHs, in order to better understand their real potential.^7^, ^20^, ^21^

In 2018 Zheng et al., comparing 0.5% topical timolol to laser therapy in treating IHs, assessed that topical timolol is not only more effective than laser, but also safer.^7^

However, data concerning the differences between topical beta blockers and laser therapy (mainly PDL laser) in terms of clinical outcomes are still not conclusive, as Ying et al. in 2017 demonstrated that 595‐nm pulsed dye laser can actually lead to a faster and greater lesion improvement if compared to 0.5% topical timolol cream (four times per day).^20^

On the other hand Danarti et al. reviewed over 200 cases of IHs treated with 0.5% timolol maleate solution (two drops twice daily), 0.5% timolol gel and topical ultrapotent corticosteroids (clobetasol propionate 0.05% twice daily), demonstrating that after 6 months of therapy both timolol solution and gel resulted in a greater reduction in the size of the lesions compared to corticosteroids, while no differences were found between the two timolol formulations.^21^

### 2.4 Topical beta‐blockers in ulcerated hemangiomas

Topical beta‐blockers have been described as possible treatments for ulcerated hemangiomas, despite their defined role being still under investigation.^1^

As a matter of fact, although being considered generally safe, topical timolol is indeed related to a higher risk of absorption when administered on ulcerated areas, thus leading to the legitimate concern for possible systemic side effects.^1^, ^22^

However, despite its potential risks, topical timolol has demonstrated a great clinical impact on ulcerated IHs. In fact, 9 cases of ulcerated hemangiomas were successfully treated with topical beta‐blockers, among which 6 cases were treated with 0.5% topical timolol (ophthalmic solution), 2 cases with 1% topical propranolol (in an oil based cream) and pulsed‐dye laser, and 1 case with 0.2% brimonidine and 0.5% timolol eye drops. Therefore, all cases treated with 0.5% timolol (including the single case treated with 0.2% brimonidine and 0.5% timolol eye drops) showed a fast onset of healing within 1 week, and complete healing within 2 weeks up to 1.5 months, with no adverse effects.^23^ The 2 cases treated with topical propranolol and pulsed‐dye laser showed a rapid healing within 3 and 6 weeks.^23^

These data are even more important when compared to those regarding the use of oral propranolol in the treatment of ulcerated hemangiomas. As a matter of fact, 4 out of 33 ulcerated hemangiomas treated with 2 or 3 mg/kg/die oral propranolol experienced ulcer recurrence after stopping treatment, while the previously reported cases of ulcerated IHs treated with topical beta‐blockers (both 0.5% timolol and 1% propranolol, as illustrated above) showed no recurrence at up to 18 months after therapy cessation.^23^

**FIGURE 1:**
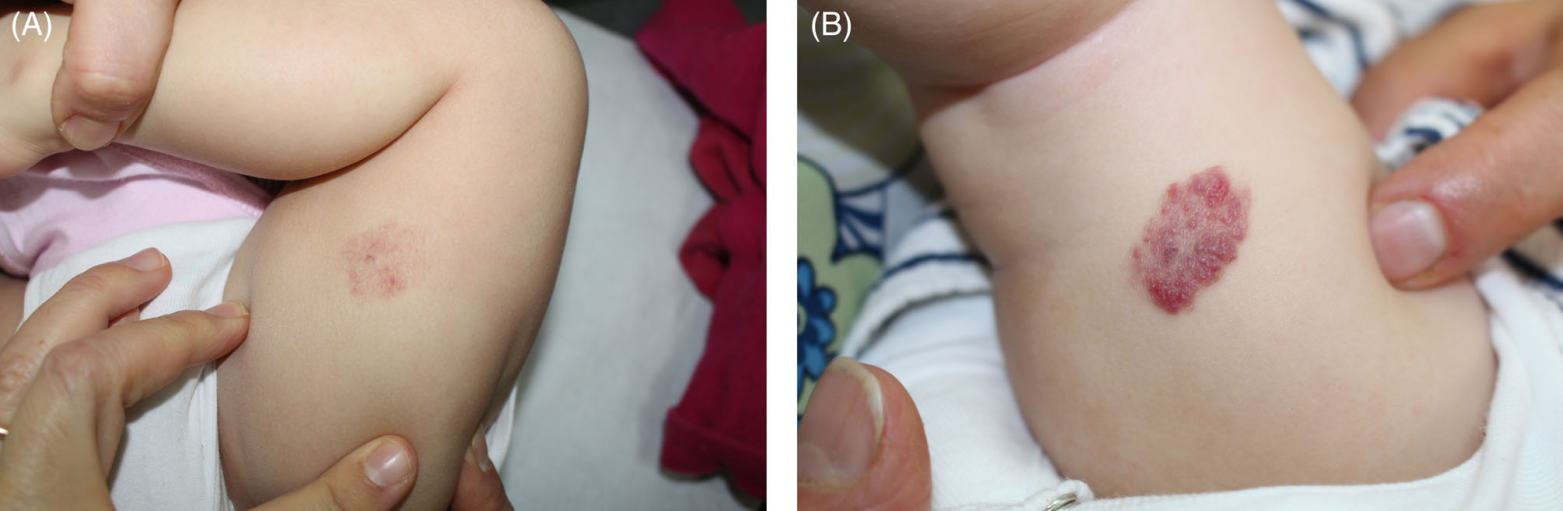
Superficial infantile hemangioma of the leg of a 4 months‐old baby. Before treatment (A) and after 9 months of treatment (B) with propranolol 1% in petrolatum cream. (Parents signed consent form for publication of clinical picture)

In conclusion, topical beta‐blockers are a valid therapeutic option in treating IHs, especially if superficial (Figure 1). Not only have they proven safe and efficient, with no differences emerging between topical timolol and topical propranolol, but they also resulted as clinically successful as oral beta‐blockers. However, many questions still require definitive answers, as no conclusive data are available regarding the best vehicle of administration, posology and their true role in treating ulcerated hemangiomas. Further studies are therefore still necessary to clarify these aspects.

## 3 BETA‐BLOCKERS IN PYOGENIC GRANULOMA AND NAIL PARONYCHIA

PG or lobular capillary hemangioma, is a rapidly growing benign vascular tumor that more often presents in children less than 5 years of age. It can arise spontaneously or may be induced by local trauma or drugs at sites of injury or within a capillary malformation. It develops most commonly on the head, neck, and upper extremities (on the skin or mucosae) with a slight predominance in females.^22^

Clinical presentation consists of a small, friable, red papule, or nodule. Satellitosis has been observed in pediatric PG. Histologically, PG is composed of capillaries and venules with plump endothelial cells separated into lobules by fibromyxoid stroma.^22^

If untreated, these lesions most often persist, may enlarge and continue to bleed intermittently. Treatment is often required due the risk of ulceration and bleeding. Treatment modalities include intralesional bleomycin, corticosteroids, ethanol, topical treatment with phenol, imiquimod 5%, laser therapy, curettage, electrocautery, radiosurgery, cryosurgery, and surgical excision. High recurrence rates limit other therapies, such as topical silver nitrate and cryotherapy.^22^ Surgical procedures may be traumatic, general anesthesia is sometimes dangerous and the surgical scar is evident, but this solution has the advantage of histological confirmation.

Topical beta‐blockers are now an excellent noninvasive option for the treatment of PG. Their use can postpone or obviate surgical treatments, especially in children, in which they are the first line treatment. They can also be useful in large lesions to reduce the size of PG or postpone or obviate surgery. The only limitation of beta‐blockers is the impossibility of performing histological examination.^24^

Beta‐blockers inhibit VEGF, decreasing angiogenesis and inducing vasoconstriction and apoptosis of endothelial cells. PGs express approximately half as many beta‐receptors as infantile hemangiomas, which may explain the less robust response of PG to beta‐blockers.^24^

In the past 5 years, several case reports, case series, prospective and retrospective studies have supported the use of topical beta‐blockers such as timolol and propranolol in PG treatment.^22^, ^24^, ^25^, ^26^, ^27^, ^28^, ^29^ Timolol maleate in 0.5% gel formulations is the most widely used topical beta‐blocker for PG.^25^ A helpful case series was described by Gupta^22^ in 2016, reporting 10 patients (aged 15–50 years) treated with 0.5% timolol maleate ophthalmic solution four times a day, obtaining complete resolution in 5 cases (as from 3 days and within 24 days), partial resolution in 2 patients, and no response in 2 patients. Also periocular granuloma was treated with timolol 0.5% gel twice a day in 4 children in the work by Oke et al., achieving complete resolution and no recurrence for at least 3 months.^26^

In literature, PG treated with a sequence of multiple treatment modalities has been described. For example, Chiriac et al. treated a healthy 2‐year old boy with a red pedunculated nodule on the left palpebral area and a 13‐month old girl with timolol 0.1% in occlusive dressing twice daily for 2 weeks, followed by 70% trichloroacetic acid (TCA) at 7‐day intervals, obtaining complete resolution.^27^

Since 2017 topical propranolol has also been used in the treatment of PG. In a recent open label prospective study Neri et al. treated 22 patients with topical propranolol 1% ointment twice daily under occlusion (hydrocolloid dressing), with complete response in most cases (59% complete regression after an average of 9 and a half weeks, and none of the regressed PG relapsed within 2 years of follow up).^28^ According to this study a hydrocolloid dressing may increase the penetration and efficacy of the treatment. Mashiah et al. demonstrated that propranolol 4% without occlusion for 6 and a half weeks brought about complete or almost complete regression in 72% of patients (18 children).^22^ In this study they demonstrated a good safety profile of propranolol 4 gel%, with no side effects and only skin irritation in one patient. Indeed, the higher propranolol concentration in gel eliminates the need for occlusion and also demonstrates that the treatment duration is probably related to the localization and size of the lesions.

Recently, topical beta‐blockers such as propranolol 1% cream^30^ or timolol 0.5% gel^31^ or betaxolol 0.25% eye drops^32^ have been used as a new treatment for drug‐induced nail paronychia.

These painful lesions are PG‐like lesions that occur on the nails of hands and feet in 10%–15% of patients treated with epidermal growth factor receptor inhibitors (EGFR‐I). EGFR‐I (cetuximab, panitumumab, erlotinib, gefitinib, lapatinib, afatinib, and osimertinib) are a class of targeted therapies approved for the treatment of several types of solid organ tumors (non‐small cell lung cancer, colorectal, breast, head and neck, and pancreatic cancer).^31^

Excellent or partial responses have been reported in a literature review,^31^ but only in Piraccini's work with propranolol cream was there a difference between a good response on the fingernails and no effect on the toenails. The authors suggest that this difference is probably to a possibly inadequate vehicle that was not able to penetrate the thicker skin of the feet, or to a low drug concentration.^30^

In summary, beta‐blockers have proven to be safe and well tolerated in the treatment of PG mostly for small, superficial infantile PG and also for PG‐like lesions induced by EGFR‐I. Larger randomized studies are needed to determine the best regimen and fully delineate the safety and efficacy of topical beta‐blockers. Adverse effects and systemic absorption appear to be negligible, although further studies are needed to determine maximal dosage.

## 4 BETA‐BLOCKERS IN KAPOSI SARCOMA TREATMENT

KS is an unusual vascular tumor, probably of lymphatic lineage, showing aberrant endothelial and mesenchymal differentiation.^33^ To date, an effective, low‐risk treatment for KS has not been found. Topical treatment would be ideal for patients with disease confined to the skin, since it may slow disease progression for several years and pose lower risks of systemic side effects. In fact, some topical agents have been used, including retinoids, imiquimod, rapamycin, and recently timolol maleate solution, with varying results.^33^, ^34^

Molecular studies have shown that KS lesions are dependent on beta‐adrenergic signaling for the reactivation of human herpes virus‐8, which could explain why beta‐blockers can reduce KS proliferation by suppressing cAMP and protein kinase A signaling pathways.

To date, 12 patients with KS treated with topical timolol have been described in the literature.^33^, ^34^, ^35^ All the patients (except one)^33^ responded rapidly to treatment in a time period between 5 and 24 weeks; different timing responses can be possibly related to lesions size.

Of the patients who experienced complete resolution, disease recurrence monitoring was not reported for 4 patients, while in the remaining patients no recurrence was reported at a mean follow‐up of 12.3 months. There were no differences in response rate between immunocompetent and immunosuppressed patients.

Most patients have been treated with 0.5% topical timolol but recently it has been reported that lower concentrations (0.1% instead of 0.5%) might also be efficacious.^36^ The main advantages of topical timolol in KS treatment are low cost, ease of administration and minimal adverse effects; all these are key elements since KS can be endemic in equatorial areas, and are very frequent in patients with comorbidities.

## 5 BETA‐BLOCKERS AND WOUNDS

Since the positive effect of oral propranolol in the adjuvant therapy of severe burns was first described, beta‐blockers are increasingly being used for management of chronic nonhealing wounds. Beta‐blockers can promote wound angiogenesis, keratinocytes migration through ERK phosphorylation and the inhibition of cellular proliferation, as well as myofibroblast density, collagen deposition; all these effects can improve wound re‐epithelialization. ^37^

Animal studies have shown that lesions treated with topical beta‐blockers have a higher EGF expression, epidermal, and dermal regeneration compared to controls. ^37^ These findings have led to clinical trials using beta‐blockers to improve healing of wounds.

Topical beta‐blockers were successfully used to treat chronic venous leg ulcers and wounds.^37^, ^38^, ^39^ A prospective non randomized single‐center study by Thomas et al.^37^ showed that the patients treated with 0.5% topical timolol maleate had a significantly higher ulcer healing rate (61.79%) compared with the control group (29.62%) after 12 weeks of treatment. Rai et al.^39^ conducted a randomized controlled study comparing 10 patients with chronic venous leg ulcers treated with 0.5% topical timolol ophthalmic solution to 10 patients treated with saline dressings. They demonstrated that patients in the timolol group (86.80%) had a significant reduction in the size of the ulcer as compared to the saline group (43.82%) after 4 weeks of treatment. There were no side effects and the treatment was well tolerated.

The usefulness of topical timolol was also demonstrated in addition to autologous adipose mesenchymal stem cell‐enriched high‐density lipoaspirate in the management of a patient with chronic ulcers.^40^

Wound improvement was also described in two children with junctional epidermolysis bullosa treated with 0.5% timolol maleate ophthalmic solution; 100% and 80% healing was achieved after 3 and 8 weeks of treatment. The authors suggest that only small areas should be treated, considering its possible systemic absorption.^41^

## 6 OTHER USES OF TOPICAL BETA‐BLOCKERS

The molecular rationale for the use of beta‐blockers in neutrophil‐mediated inflammatory skin diseases such as pyoderma gangrenosum^42^ is that propranolol enhances IL‐8, induces neutrophil chemotaxis and reduces the release of reactive oxygen species after immune complex stimulation.

From this wound‐healing standpoint, the effects of topical timolol on acute surgical wounds have been tested in six patients, demonstrating that topical timolol application could improve the overall cosmesis of acute surgical wounds, decreasing vascularity compared to control.^43^

A similar positive effect has been demonstrated in the management of graft versus host disease‐associated angiomatosis^44^ and in the reduction of persistent granulation tissue in a patient with severe hidradenitis suppurativa.^45^ Since granulation tissues are highly vascular and their histological aspects are very similar to hemangiomas, topical beta‐blockers act, and achieve their clinical effects through endothelial cell apoptosis.

The vasoconstrictor effect of beta‐blockers on vessels has been demonstrated by its successful topical use in patients with glucocorticoid‐induced skin telangiectasia,^46^ showing a significant decrease in erythema and telangiectasia. On the other hand, in a study^47^ on five patients with hereditary hemorrhagic telangiectasia, no significant changes were noted after 6 months of 0.5% timolol ophthalmic solution treatment; the authors attributed the therapy failure to the low penetration of the product utilized (ophthalmic solution, not gel).

## 7 CONCLUSIONS

The present review shows that topical beta‐blockers are a safe and valid therapeutic option in the treatment of various cutaneous diseases (Table 1). Side effects, such as irritation,^48^ redness, and scaling, are mainly restricted to the application site, while few systemic adverse effects have been reported.^49^, ^50^

**TABLE 1:** Topical beta‐blockers off‐label use in dermatologic diseases

Disorder	Preferred topical beta blocker	Most widely used concentration	Frequency of administration	Treatment duration
Infantile hemangiomas (IHs)	Timolol^16^	0.5%	Twice daily	6–9 months
	Propranolol^12^	1%	Twice daily	6–9 months
Pyogenic granuloma	Timolol^27^	0.5%	Twice daily	2 months
	Propranolol^28^, ^29^, ^30^	1%–4%	Twice daily	2 months
Nail paronychia	Timolol^31^	0.5% (under occlusion)	Twice daily	1 months
Kaposi sarcoma	Timolol^33^, ^34^, ^35^, ^36^	0.5% (some reports with 0.1%)^73^	Twice daily	3 months
Wound	Timolol^37^, ^38^, ^39^, ^40^	0.5% (1 drop/2 cm)	Once daily	3 months

Further studies and randomized trials may contribute to reinforce the role of topical beta‐blockers in the dermatological armamentarium.

## CONFLICT OF INTEREST

The authors declare no potential conflict of interest.

## AUTHOR CONTRIBUTIONS

Conceptualization: Filoni Angela; Methodology: Filoni Angela, Bonamonte Domenico; Formal analysis and investigation: Filoni Angela, Bonamonte Domenico; Writing–original draft preparation: Filoni Angela, Ambrogio Francesca, De Marco Aurora; Writing–review and editing: Filoni Angela, Bonamonte Domenico, Pacifico Alessia; Supervision: Angela Filoni, Bonamonte Domenico, Pacifico Alessia. All the authors approve the final submitted version of the manuscript.

## Data Availability

Data sharing is not applicable to this article as no new data were created or analyzed in this study.
